# Viewing research assessment, the academic reward system, and academic publishing through the power/knowledge lens of Foucault

**DOI:** 10.3389/frma.2023.1179376

**Published:** 2023-08-29

**Authors:** Timothy D. Bowman

**Affiliations:** School of Information Studies, Dominican University, Chicago, IL, United States

**Keywords:** research assessment, academic reward system, academic publishing, Foucault, power/knowledge

## Abstract

The academic research assessment system, the academic reward system, and the academic publishing system are interrelated mechanisms that facilitate the scholarly production of knowledge. This article considers these systems using a Foucauldian lens to examine the power/knowledge relationships found within and through these systems. A brief description of the various systems is introduced followed by examples of instances where Foucault's power, knowledge, discourse, and power/knowledge concepts are useful to provide a broader understanding of the norms and rules associated with each system, how these systems form a network of power relationships that reinforce and shape one another.

## 1. Introduction

This paper considers Foucault's concepts of power, knowledge, discourse, and power/knowledge as a lens to view the power relationships between research assessment, the academic reward system, and academic publishing. Using a Foucauldian lens, it is argued that these concepts can provide insights into the nature of the scholarly production of knowledge.

The assessment of academic research, which traditionally has focused on metrics relating to citations, authorship, and acknowledgments, is influenced by many sources including scholars, the academic publishing system, funding agencies, and by variations of academic reward system requirements across the world. Using Foucault's concepts, which examines the ways in which knowledge is created, disseminated, and validated, one can gain insight into how power-knowledge functions through the various practices of research assessment, the academic reward system, and academic publishing.

Research assessment, the academic reward system, and academic publishing are important components of knowledge production. According to Foucault, power is not only the result of individual agents' powers over one another, but a dispersed and ubiquitous force that is part of the dissemination and production of knowledge itself. Thus, those who are defining what knowledge is and how it is being disseminated and produced can be viewed through the lens of Foucault's framework.

Within the academic reward system, power-knowledge operates through various criteria used to evaluate scholarship and through the awarding of research funding, which in turn may shape the types of research that are prioritized and supported. When one considers academic publishing and power-knowledge, the decisions of publishers, editors, and peer reviewers can shape the types of scholarly output that are published and codified within the academic community. Finally, looking at research assessment one can find that power-knowledge is operating through the various rankings and metrics used to evaluate scholarly output and by the role of funding agencies in shaping research policy.

The evaluation of research assessment by scholarly output is based on various criteria including quality and impact and has a significant influence on funding distribution and other resource distribution. Often the academic reward system is based on an evaluation of scholarly output, teaching, and service. Academic publishing is closely tied to both the academic reward system and research assessment. Typically, publications in journals deemed high-impact or A-list journals are equated with academic success, which can lead to career advancement within the academic reward system. Similarly, academic publishing is an important factor in research assessment as research excellence is typically measured by the impact and quality of scholarly output. These three systems are interlinked and reinforce one another, which, in turn, shapes Science.

Scholars can gain better insight into how power is being applied within research assessment, the academic reward system, and academic publishing by examining the ways in which power-knowledge operates within these systems. A better understanding of this operation can lead to challenge, change, and transformation.

## 2. Foucault's power, knowledge, discourse, and power/knowledge

Foucault argued that knowledge is possible only through networks of power relationships (or systems), which allows knowledge to be created. As Townley (2019, p. 519) notes, “Foucault has shown how what counts as truth depends on, or is determined by, the conceptual system in operation.” Foucault (1980, p. 94) himself noted that “Power is not something that is acquired, seized or shared, something one holds on to or allows to slip away.” Instead of something seized or shared, Foucault argued that power is relational and can be revealed through practice at any level within systems (or networks)—it is not associated with a specific agent. His notion of power does not question who, where, or what has power, but the “how” of power. Foucault (1991, p. 52) contended that “it is not possible for power to be exercised without knowledge; it is impossible for knowledge not to engender power.” He (Foucault, 1978, p. 95) argued that “there is no power that is exercised without a series of aims and objectives.” He added to this in his *History of Sexuality Volume 1: An Introduction* (1978) when he discussed “normalizing power” as the ways in which power operates in society. This normalizing power operates via networks of discourses, technologies, and institutions, which both produces and enforces normative standards of behavior while at the same time disciplining and ostracizing those who do not attend to these norms.

As Haugaard (2022, p. 359) notes, one interpretation of Foucault's idea of power/knowledge is that “[i]n everyday life, we encounter what appears as the natural order of things. This natural order is a form of practical commonsense, which is often socially constructed as the right thing to do in social situations.” This commonsense, then, is the power/knowledge that allows everything to appear as “right” at the time, but the insight using Foucault's concepts is that this appearance of “rightness” has not always been so. With regards to power, Segev (2019) noted that Foucault “famously described how institutions disseminate certain types of knowledge as a control mechanism.” Foucault's concepts, including power, knowledge, and power/knowledge, have been used widely in the literature including to examine children's participation (Gallagher, 2008), the study of management and organizations (Knights, 1992), human resource management (Townley, 2019), Australian higher education (Marginson, 1997), academia (Gonzales and Núñez, 2014), and other domains.

In addition to power, knowledge, and power/knowledge, Foucault's concept of discourse examines the knowledge production within social and cultural systems. Dergisi (2022, p. 25) argued that according to Foucault, “[d]iscourse can only be understood through the institutions and power that produce it, and it is produced through exclusion or control.” Thus, this idea of discourse implies that social and cultural systems can be examined through the ways in which they produce, disseminate, and legitimize knowledge. Garrity (2010, p. 202) notes that Foucault's discourse “can be thought of as a practice; discourse crosses the theory–praxis divide by understanding (discursive) knowledge as a social practice—as doing something.” In addition, Boulton et al. (2022, p. 2) state that “a discourse is both a composite of and a repository for societal norms.” By looking at discourse in this way, one can examine how, within an academic context, discourse shapes the production and dissemination of knowledge by defining what counts as legitimate knowledge and who has the authority to produce and disseminate it. Several studies have used, what is framed as, Foucauldian discourse analysis (FDA) to examine power/knowledge in various contexts including societal values surrounding children and digital rights in the UK (Hope, 2015), media reporting of nurses as heroes during COVID-19 (Boulton et al., 2022), healthy eating information and advice (Mackenzie and Murray, 2021), social media and educational policy (Sam, 2019), and positive psychology in the workplace (McDonald et al., 2021).

Deacon (2006, p. 184) wrote that, according to Foucault, “[u]niversities, like schools, are multifaceted amalgamations of economic, political, judicial and epistemological relations of power… [universities] will become increasingly important politically, because they multiply and reinforce the power-effects of an expanding stratum of intellectuals.” If one were to use Foucault's concept of discourse and power/knowledge to examine the academic reward system, a researcher could view the academic reward system as a discourse that defines what types of research are rewarded and valued within academia. With regards to research assessment, the assessment system is a discourse that shapes how research is funded and evaluated. Finally, academic publishing is a discourse that defines how research is disseminated and what is considered legitimate research. Because, as Foucault argues, discourses are not neutral or objective but instead shaped by power relations. One can gain insight into the power relations that exist within, and across, these academic systems.

## 3. Research assessment system

Research assessment is the evaluation of research output by funding agencies, universities, and other stakeholders. An evaluation typically examines the impact and quality of research output, as well as the fit with research objectives and priorities. At a broader level of assessment, Gonzales and Núñez (2014) argued that we are living in the “ranking regime” era. This ranking regime entails the ranking of post-secondary education by government systems, metrics created by scholars, and commercial entities such as *US News and World Report*. These various entities “define what excellent higher education, valuable knowledge, or at the grandest level, ‘world-class universities' are made of” (Gonzales and Núñez, 2014, p. 3). Others have also examined the effects of the ranking regime on post-secondary educational institutions including organizational reputation (Bastedo and Bowman, 2010), reputation, status signals, and impact (Bowman and Bastedo, 2009), and competition (Ehrenberg, 2003). Bloch (2004, p. 5) argued that, when examining the 2002 National Research Council's report titled *Scientific Research in Education*, the discourse of “hard science” found in the report must “be viewed as having created a regime of truth about good educational research that is based in its own cultural, political, economic, and social context… the report is not context free.” The ranking regime and the discourse surrounding these mechanisms are not, as Bloch (2004) noted, context free. From a Foucauldian perspective, power operates not just through blatant actions of force, but also via more indirect forms of normalization and control. Within the academic community, these rankings and other external evaluations can shape the perception and value of universities within a larger social context.

Recently some schools, departments, and universities have decided to stop providing *US News and World Report* with data (Svrluga and Anderson, 2023), effectively removing themselves from the power rankings. When looking at this from a Foucault perspective, one might consider this a pushback against external forms of evaluation and surveillance. These ranking systems can shape the ways in which universities, schools, and departments are perceived, which demonstrates the power relations at play within academia. Through these rankings we can find the “how” of power. Heather Gerken, the Yale Law School Dean, was quoted as saying “We have never paid attention to U.S. News and its rankings… What we are talking about are the values of legal education and the profession” (Hartocollis, 2023). In the prior quote, Dean Gerken's statements reflect a pushback against *US News and World Report's* external evaluation and the establishment of ranking norms and the legitimizing of specific values at the expense of other values.

In another example, scholars have attempted to liberate themselves from common and accepted practices used in research assessment, which Foucault deemed normalizing power, through the introduction of The San Francisco Declaration on Research Assessment (DORA)^1^ and the Leiden Manifesto (Hicks et al., 2015). DORA was introduced in 2012 to “improve the ways in which the outputs of scholarly research are evaluated.” DORA, to date, has received 22,716 signatures from individuals and organizations across 159 countries (DORA, n.d.). The authors of DORA noted that the “recommendations are aimed at funding agencies, academic institutions, journals, organizations that supply metrics, and individual researchers.” The Leiden Manifesto (Hicks et al., 2015) was intended to address the “the pervasive misapplication of indicators to the evaluation of scientific performance.”

Each of these documents are intended to challenge the normalization of metrics and other forms of evaluation used in research assessment while both promoting alternate approaches to evaluate the impact and quality of research. Using a Foucauldian lens, both acts can be considered as arbitrations in the academic research assessment power/knowledge relations. Within the academic community, these metrics and other external evaluations can shape the perception and value of research within a larger social context. Both DORA and the Leiden Manifesto are challenging the norms of metrics use and asserting the importance of more diverse forms of knowledge production and dissemination.

## 4. Academic publishing system

Academic publishing involves the publishing of academic research and scholarship output in various formats including in academic journals as articles, as books, or as theses. Peer review is principal to most academic publishing, which entails peer scholars in a domain determining if the submission is quality enough to be published. Peer review feedback can result from single-blind, double-blind, or open forms, which is given before the scholarship is published. The process of academic publishing involves two broad stages: (1) peer review and one or more rounds of modification, and (2) editing, typesetting, and printing/online an official release. In a critique of the academic publishing industry, Beverungen et al. (2012) explained the academic publishing model as:

In the first moment, publishers claim the intellectual property rights for knowledge produced in universities even though they do not recompense the producers of that knowledge for its production. In the second moment, the publishers sell, at a massively inflated price, this same knowledge back to the universities so that its producers can deploy that knowledge in further research and in teaching (p. 4).

Sugimoto and Larivière (2018, p. 119) argued that the control of scholarship and research measurement by for-profit publishers is a “perversion of the system.”

Academic publishing is embedded in larger power relations that influence the direction and content of academic research. Academic publishers can be subjected to various influences from actors outside the publishing industry including universities, funding agencies, the government, as well as other social and political contexts. As Bristow (2021, p. 4) notes, “[t]o put it in Foucauldian terms, right from the outset scholarly periodicals grew into what can be understood as capillaries and conduits of power-knowledge (Foucault, 1980, 1991) constitutive of the Enlightenment and then post-Enlightenment science.” In addition to this, Roth (2002, p. 216) argues that “it is apparent that editors, reviewers, tenure committees, and others who make decisions about authors' publications (or publication records) are in strategic positions from which they exercise power.” Roth (2002) goes on to note that journal editors can exercise and gain power by ensuring the collaboration of all those involved in the peer review process—editors are a vital component of the system and reflect the power relationship of which they are afforded. The gatekeeping role undertaken by journal editors allows for the power to decide which research is deemed worthy of publication and dissemination.

Just as journal editors are important actors in the power/knowledge relations shaping the academic community, peer reviewers also play a critical role in identifying scholarly output of sufficient quality and relevance. Peer reviewers also take on the gatekeeping role. Hackett and Chubin (2003, p. 7) argued that peer review could be considered as a boundary process, “in the sense that it spans the boundaries of several social worlds.” The authors also noted that peer reviewers utilize various procedures, norms, rules, and culture that coalesce during the review process. Using a Foucauldian perspective, one can surmise that peer reviewers, like journal editors, are key actors associated with determining which research holds value and subsequently published and disseminated.

## 5. Academic reward system

The academic reward system is made up a variety of structures, technologies, and discourses, which produce and reinforce academic achievement and success. Within the academic reward system, power operates through norms and standards that shapes and regulates academic identity and behavior. These norms and standards can include various forms of evaluation including citation counts, publication numbers, the amount of grant funding, or subsequent forms evaluation such as tenure and promotion. However, just as with research assessment and academic publishing, the academic reward system is subject to a variety of influences including from universities, funding agencies, and broader political or social factors.

One key area of inconsistency is the area of tenure and promotion. While some institutions place emphasis on scholarly productivity, others may prioritize teaching and service (Schimanski and Alperin, 2018; Niles et al., 2020; Rice et al., 2020). This inconsistency highlights the ways in which academics must tailor their activities and priorities based on the demands of their university, school, or department, which influences the types of knowledge that are valued and produced. Evaluations (Alperin et al., 2019; McKiernan et al., 2019) of promotion and tenure documents from the United States revealed inconsistencies between the expectations and evaluations of tenure-track faculty. In United States Pharmacy schools, Snider et al. (2021) found that tenure and promotion documents often were not specific when defining requirements. In a letter to Science, Htun (2020) discussed the impact that COVID-19 had on tenure-track faculty and recommended three adjustments to the tenure and promotion evaluation process: (1) candidates could choose the 6 most productive years to be used for their tenure package, (2) candidates are required to submit a COVID-19 impact statement, and (3) tenure and promotion evaluators should utilize qualitative and holistic assessments in addition to quantitative metrics. When surveying administration across 115 Carnegie R1 classified universities, Bales et al. (2019) found that research continues to be the most important criteria for granting tenure and promotion. Corneile et al. (2019) found that faculty who were women of color experienced a higher burden of teaching, service, and mentorship. De Los Reyes and Uddin (2021) argued that the means in which historically underrepresented faculty are evaluated for tenure and promotion are built upon biased evaluation metrics. From a power/knowledge perspective, this inconsistency can have an impact on the academic community as it shapes the knowledge that is valued and produced, as well as determining the availability of resources provided to early career academics.

The term, or dictate, “publish-or-perish” has been bandied around for decades in the academic world as it relates to the push for scholars to publish frequently in journals considered to be the most impactful in a specific domain, as rightly or wrongly determined by various assessment metrics, to earn tenure or promotion within a given time period (typically 6 years). As De Rond and Miller (2005, p. 322) note, “[d]uring the past four decades, the publish or perish principle appears to have become the way of life in academia.” Rawat and Meena (2014, par. 2) argue that “he emphasis on publishing has decreased the value of the resulting scholarship as scholar must spend time scrambling to publish whatever they can manage, rather than spend time developing significant research agenda.” From a Foucauldian perspective, publish-or-perish implies a normalization within the academic reward system. It signifies that the production and dissemination of knowledge through publishing is a normalization of power, and that failure will result in negative consequences. As Madikizela-Madiya (2023) writes, the pressures of publish-or-perish “side-lines the actual and relevant purpose of research, which is to develop new knowledge or extend the existing knowledge toward developing solutions for society.”

## 6. Discussion

This work is an attempt to demonstrate the potential usefulness of Foucault's concepts and frameworks to investigate the power relationships, as power, knowledge, discourse, and power/knowledge, of the research assessment system, the academic publishing system, and the academic reward system. These systems are foundational to the academic community and demonstrate various normalizations of power, which dictate how academic knowledge is produced and disseminated within the academic community. Each of these systems are interrelated in terms of power relationships; they form a network of reinforcement and shape each other.

Examples of power relationships were discussed, and previous research was used to highlight the power distribution amongst actors in the research assessment system, the academic publishing system, and the academic reward system. Within the research assessment system, standards are created that regulate behavior and identity. Whereas, the academic reward system incentivizes other types of behavior and knowledge production, reinforcing the standards of the research assessment system. The academic publishing system also reinforces the normalization of power through gatekeepers and reinforces those standards of both the research assessment system and the academic reward system. They operate as mechanisms of power.

Future work should consider implementing Foucauldian discourse analysis (FDA) to examine power/knowledge within, and across, these various systems.

## Author contributions

The author confirms being the sole contributor of this work and has approved it for publication.

## Footnotes

^1^ https://sfdora.org/read/
